# The European Nucleotide Archive in 2020

**DOI:** 10.1093/nar/gkaa1028

**Published:** 2020-11-11

**Authors:** Peter W Harrison, Alisha Ahamed, Raheela Aslam, Blaise T F Alako, Josephine Burgin, Nicola Buso, Mélanie Courtot, Jun Fan, Dipayan Gupta, Muhammad Haseeb, Sam Holt, Talal Ibrahim, Eugene Ivanov, Suran Jayathilaka, Vishnukumar Balavenkataraman Kadhirvelu, Manish Kumar, Rodrigo Lopez, Simon Kay, Rasko Leinonen, Xin Liu, Colman O’Cathail, Amir Pakseresht, Youngmi Park, Stephane Pesant, Nadim Rahman, Jeena Rajan, Alexey Sokolov, Senthilnathan Vijayaraja, Zahra Waheed, Ahmad Zyoud, Tony Burdett, Guy Cochrane

**Affiliations:** European Molecular Biology Laboratory, European Bioinformatics Institute, Wellcome Genome Campus, Hinxton, Cambridge CB10 1SD, UK; European Molecular Biology Laboratory, European Bioinformatics Institute, Wellcome Genome Campus, Hinxton, Cambridge CB10 1SD, UK; European Molecular Biology Laboratory, European Bioinformatics Institute, Wellcome Genome Campus, Hinxton, Cambridge CB10 1SD, UK; European Molecular Biology Laboratory, European Bioinformatics Institute, Wellcome Genome Campus, Hinxton, Cambridge CB10 1SD, UK; European Molecular Biology Laboratory, European Bioinformatics Institute, Wellcome Genome Campus, Hinxton, Cambridge CB10 1SD, UK; European Molecular Biology Laboratory, European Bioinformatics Institute, Wellcome Genome Campus, Hinxton, Cambridge CB10 1SD, UK; European Molecular Biology Laboratory, European Bioinformatics Institute, Wellcome Genome Campus, Hinxton, Cambridge CB10 1SD, UK; European Molecular Biology Laboratory, European Bioinformatics Institute, Wellcome Genome Campus, Hinxton, Cambridge CB10 1SD, UK; European Molecular Biology Laboratory, European Bioinformatics Institute, Wellcome Genome Campus, Hinxton, Cambridge CB10 1SD, UK; European Molecular Biology Laboratory, European Bioinformatics Institute, Wellcome Genome Campus, Hinxton, Cambridge CB10 1SD, UK; European Molecular Biology Laboratory, European Bioinformatics Institute, Wellcome Genome Campus, Hinxton, Cambridge CB10 1SD, UK; European Molecular Biology Laboratory, European Bioinformatics Institute, Wellcome Genome Campus, Hinxton, Cambridge CB10 1SD, UK; European Molecular Biology Laboratory, European Bioinformatics Institute, Wellcome Genome Campus, Hinxton, Cambridge CB10 1SD, UK; European Molecular Biology Laboratory, European Bioinformatics Institute, Wellcome Genome Campus, Hinxton, Cambridge CB10 1SD, UK; European Molecular Biology Laboratory, European Bioinformatics Institute, Wellcome Genome Campus, Hinxton, Cambridge CB10 1SD, UK; European Molecular Biology Laboratory, European Bioinformatics Institute, Wellcome Genome Campus, Hinxton, Cambridge CB10 1SD, UK; European Molecular Biology Laboratory, European Bioinformatics Institute, Wellcome Genome Campus, Hinxton, Cambridge CB10 1SD, UK; European Molecular Biology Laboratory, European Bioinformatics Institute, Wellcome Genome Campus, Hinxton, Cambridge CB10 1SD, UK; European Molecular Biology Laboratory, European Bioinformatics Institute, Wellcome Genome Campus, Hinxton, Cambridge CB10 1SD, UK; European Molecular Biology Laboratory, European Bioinformatics Institute, Wellcome Genome Campus, Hinxton, Cambridge CB10 1SD, UK; European Molecular Biology Laboratory, European Bioinformatics Institute, Wellcome Genome Campus, Hinxton, Cambridge CB10 1SD, UK; European Molecular Biology Laboratory, European Bioinformatics Institute, Wellcome Genome Campus, Hinxton, Cambridge CB10 1SD, UK; European Molecular Biology Laboratory, European Bioinformatics Institute, Wellcome Genome Campus, Hinxton, Cambridge CB10 1SD, UK; European Molecular Biology Laboratory, European Bioinformatics Institute, Wellcome Genome Campus, Hinxton, Cambridge CB10 1SD, UK; European Molecular Biology Laboratory, European Bioinformatics Institute, Wellcome Genome Campus, Hinxton, Cambridge CB10 1SD, UK; European Molecular Biology Laboratory, European Bioinformatics Institute, Wellcome Genome Campus, Hinxton, Cambridge CB10 1SD, UK; European Molecular Biology Laboratory, European Bioinformatics Institute, Wellcome Genome Campus, Hinxton, Cambridge CB10 1SD, UK; European Molecular Biology Laboratory, European Bioinformatics Institute, Wellcome Genome Campus, Hinxton, Cambridge CB10 1SD, UK; European Molecular Biology Laboratory, European Bioinformatics Institute, Wellcome Genome Campus, Hinxton, Cambridge CB10 1SD, UK; European Molecular Biology Laboratory, European Bioinformatics Institute, Wellcome Genome Campus, Hinxton, Cambridge CB10 1SD, UK; European Molecular Biology Laboratory, European Bioinformatics Institute, Wellcome Genome Campus, Hinxton, Cambridge CB10 1SD, UK; European Molecular Biology Laboratory, European Bioinformatics Institute, Wellcome Genome Campus, Hinxton, Cambridge CB10 1SD, UK

## Abstract

The European Nucleotide Archive (ENA; https://www.ebi.ac.uk/ena), provided by the European Molecular Biology Laboratory's European Bioinformatics Institute (EMBL-EBI), has for almost forty years continued in its mission to freely archive and present the world's public sequencing data for the benefit of the entire scientific community and for the acceleration of the global research effort. Here we highlight the major developments to ENA services and content in 2020, focussing in particular on the recently released updated ENA browser, modernisation of our release process and our data coordination collaborations with specific research communities.

## INTRODUCTION

The European Nucleotide Archive (1; ENA) captures, preserves and freely presents the world's nucleotide sequence data. Launched 38 years ago, the ENA has evolved and developed through major shifts in data volume, nucleotide sequence technology and the way users utilise our services to record and conduct their research. These shifts have led particularly in recent years to significant redevelopment of our services that have culminated this year in the launch of the new ENA browser and a significant move away from a traditional quarterly release that we have conducted since the 1980s to a new continuous distribution model. The ENA provides a portfolio of services to support a broad spectrum of users in depositing and extracting a range of open access raw, assembled and annotated sequence data, as well as supporting specific collaborations with partners through our data coordination activities. The ENA is part of the International Nucleotide Sequence Database Collaboration (2; INSDC) along with our counterparts at the National Institute of Genetics’ DNA DataBank of Japan (3; DDBJ) and the United States National Center for Biotechnology's (NCBI) GenBank and Sequence Read Archive (4). Collectively the INSDC partners provide globally comprehensive coverage through mutual data exchange, collaborate on development of scientific standards and data formats, and promote the sharing of well-structured and open access sequence data.

In 2020, we have continued to develop and provide comprehensive services for the submission, archiving, discovery and retrieval of data across the range of sequencing platforms and scientific applications. In this article we will focus in particular on the development and deployment of our new ENA browser, the shift away from our traditional quarterly snapshot releases to a continuous distribution model and key highlights from our data coordination collaborations with a broad range of research communities.

## ENA CONTENT AND SERVICES

We have continued to operate and develop our portfolio of services through a range of entry points (Table 1) for data submissions, coordination, archiving, presentation and discovery of nucleotide sequence data.

**Table 1.** ENA services, description and entry points

Services	Service entry points	Description	Link to service
Data submission	Submission tools	Submission, update and data management tools	https://www.ebi.ac.uk/ena/browser/submit
Data discovery and access	ENA browser	Data discovery through array of search, filtering and data access services	https://www.ebi.ac.uk/ena
User support	Support contact form	Contact and feedback to Help desk	https://www.ebi.ac.uk/ena/browser/support
	Support documentation and training guides	Data Submission, update and discovery guidelines, training & FAQs	https://www.ebi.ac.uk/ena/browser/guides
Data coordination	Documentation and links	Introduction to programme and links to current projects	https://www.ebi.ac.uk/ena/browser/about/data-coordination

For data submission and archiving we have extended the Webin-CLI data validation and submission tool, to support direct presentation of analysis objects to allow more rapid data throughput and to add a new data type for metatranscriptomic assemblies (https://ena-docs.readthedocs.io/en/latest/submit/assembly/metatranscriptome.html). The more rapid transition times for submitted data to reach our presentation layers has been particularly important for our response to the COVID-19 pandemic. This ensures that submitted SARS-CoV-2 assemblies are rapidly available to the scientific community through both the ENA browser and presentation services, as well as the dedicated COVID-19 data portal (5; https://www.covid19dataportal.org/) to which all SARS-CoV-2 viral data is automatically routed.

To further improve the speed of data flow we have continued to modernise and improve the ENA back-end infrastructure to support the rapid growth and throughput that we continue to observe yearly. This includes specific enhancements to cross-reference services, distribution and indexing processes and their choreography and presentation for INSDC exchange to our counterpart archives at NCBI and DDBJ. With regards to enhancing the INSDC collaborative data exchange, we have implemented and deployed a new workflow that routes incoming high-volume INSDC sequence records in assembly contig sets directly to FTP presentation servers where they are immediately indexed for search and served to users directly. This change has brought a significant reduction in congestion of the relational database in which we traditionally manage all sequence records and reduced file transit and conversion times significantly. This again is a key development for our part in the COVID-19 pandemic global scientific response as viral SARS-CoV-2 records archived at our partner INSDC databases are more rapidly made available from our presentation services for direct use and integration into downstream automated services that use the ENA as a COVID-19 data feed.

**Figure 1.**
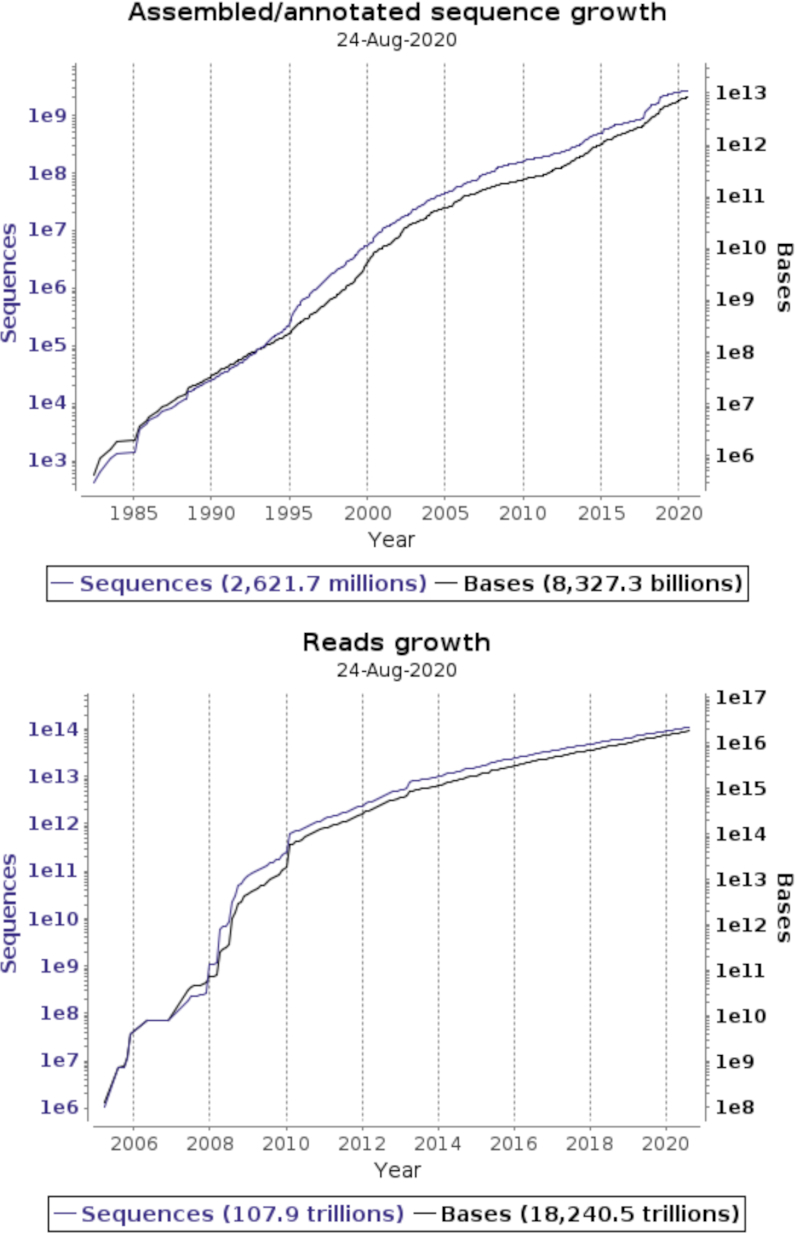
ENA Data growth of sequences and bases, for assembled/annotated sequences and read data types.

We are also now in the process of normalising sample data management into a single central EMBL-EBI service, that will include sample management of ENA direct submitted and INSDC exchanged records. The EMBL-EBI BioSamples service (6) will act as the authority for all sample records for the ENA, and we therefore over the last year been focusing on development of BioSamples data exchange with our INSDC partners and integration of sample records into the centralised EBI-Search service (7; https://www.ebi.ac.uk/ebisearch) for standardised sample use across internal EMBL-EBI archives and for external users. Our submission services have supported 2600 active data submitters from numerous countries and research areas over the last year, covering over 700 000 submissions to the ENA in 12 months, comprising of 6600 studies, over a million samples and runs, and 160,000 (meta)genome assemblies. Figure 1 shows ENA data growth of both assembled and annotated sequences and raw reads, with the totals including records from data exchange with our INSDC partners.

## SELECTED DEVELOPMENTS IN 2020

### New ENA browser, documentation and online training

The new ENA browser (https://www.ebi.ac.uk/ena) was released in *beta* in July 2019 for an extensive period of community testing and acclimatisation. We reported its array of new and updated features extensively in last year's update (1). Following positive feedback from the communities we serve we released the new browser into production in August 2020, retiring the old browser and redirecting all traffic to the new services. We have continued to improve and develop the browser's services throughout the year.

The ENA cross-reference system holds an array of key cross-references to both internal and external data archives, such as protein sequence and functional information in UniProt (8; https://www.uniprot.org/) and the ribosomal RNA sequence data records in SILVA (9; https://www.arb-silva.de/). As the popularity of the cross-referencing system has grown significantly in recent years we have added a web form for managing new cross-reference requests to capture the scope of the cross-references and the nature of the mappings that can be provided to improve the establishment of new cross reference mappings into ENA.

The new checklist browser service (https://www.ebi.ac.uk/ena/browser/checklists) provides an important component of the ENA submission model, allowing users to identify the appropriate metadata checklist and understand submission requirements, as well as assisting communities in developing their own standards through a clearer understanding of established use cases.

A key development for the effective and rapid response in assisting our userbase is our newly implemented content management system for user documentation and displayed text of our ENA browser website and presentation services. This system utilises Read-the-docs (https://readthedocs.org/) technology hosted through our own GitHub instance (https://github.com/enasequence/ena-browser-documentation) with automated synchronisation of text updates to the ENA browser through the Read-the-docs API. This means that any team member, or any user through pull requests and subsequent approval, can push text updates on GitHub that will then be automatically rendered on the ENA website without the need for a full site redeployment. To coincide with the updated content management system we have extensively updated and redrafted our website text and documentation to provide simplified guidance to our ENA presentation services. We have also developed a range of online training modules to complement the documentation (https://ena-docs.readthedocs.io/en/latest/index.html), these tutorials guide users through the whole process of data submission, data discovery and retrieval, providing data updates and a range of useful tips and tricks. The documentation includes worked examples, screenshots, code block examples and video webinars. Our support guides page (https://www.ebi.ac.uk/ena/browser/guides) lists all of the available documentation for our services.

### New release process

Since 1982 the ENA has made 143 individual releases, providing a quarterly snapshot of ENA assembled and annotated sequence data. During this time, changes to the ways in which our users access ENA data, have led us to develop a portfolio of data access tools, such as our daily FTP products (ftp://ftp.ebi.ac.uk/pub/databases/ena/) and the ENA browser API (https://www.ebi.ac.uk/ena/browser/api/), which have been offered in parallel to the traditional release for a number of years. More recently, like many ‘omics archives, we have faced growing pressure on our release process in response to significant increases in data volume from technological and economic developments, and have also seen a shift towards our newer continuous distribution services on the part of the majority of users. Our release process has remained largely unchanged for the last two decades, providing a platform from which many global services that required periodically updated public sequencing data have been developed. Last year we concluded that we would redirect full effort towards continuous distribution services and discontinue the traditional release process as part of our presentation portfolio; we thus made our last release in March 2020, release 143. It was recognised that this was a major shift in data access method for some users and we provided significant assistance to those affected through our help desk, direct support to major internal and external database consumers such as UniProt (8), SILVA (9) and RNAcentral (10), and have provided a help guide for moving away from the traditional release (https://ena-browser-docs.readthedocs.io/en/latest/help_and_guides/moving_from_release.html). This guide outlines FTP and API access to assembled/annotated sequences, guidance on how to identify data based on a last-updated timestamp and advice for establishing user-side mirroring procedures using our portfolio of other access services. No longer making an additional separate quarterly release of the assembled/annotated subset of sequences allows us to focus our resources on further developing and supporting our continuous distribution presentation products. We ourselves are now direct consumers of the ENA discovery and browser APIs through which filters can be used to replicate retrieval of ENA data changes over any range of dates using timestamp filters. The APIs can in effect be used for services to trigger local user-side snapshot release using filters on a timescale and repeat frequency that suits specific requirements; examples of this are covered in our moving away from release guide.

An additional connected change, coincident with release retirement, is deprecation of cumulative FTP files in the FTP update folders (e.g. http://ftp.ebi.ac.uk/pub/databases/ena/sequence/update/). These cumulative files tracked daily changes between release cycles and are no longer of value post-release deprecation. The separately maintained indexed datasets for ‘release’ and ‘update’ for sequence, coding and non-coding RNA records have been replaced with single new datatypes ‘sequence’, 'coding' and 'noncoding' respectively, that will be used in our API, browser and advanced search services.

### Data coordination services

In addition to our core archiving and presentation services, we have continued existing, and initiated new, work with our collaborative partners in various data coordination projects. These data coordination collaborations cover a broad range of scientific areas, and enable us to provide tailored and extensive support to particular scientific communities. A major development for 2020 has been our involvement in the development and launch of the European COVID-19 Data Platform (5; https://www.covid19dataportal.org/). This relies heavily on ENA infrastructure and data coordination tools produced under the ‘COMPARE’ project for SARS-CoV-2 viral sequence data management (11). We have in particular developed the ‘SARS-CoV-2 Data Hubs’ for the validation, sharing, analysis and publication of viral sequence data, and supported these across EMBL, EU and other member states to mobilise data and analysis. We also continue to operate a number of data coordination projects across (non-COVID-19) pathogens, livestock species, marine microbiology and biodiversity. A further highlight for the year is the launch of the European FAANG Data Coordination Centre that supports three communities in the sharing and analysis of livestock species functional genomics data and is supported by three new EU grants—GeneSwitch, BovReg and AquaFAANG—for which we provide data coordination services (12; https://data.faang.org/projects). We have initiated the EU EarlyCause project (https://earlycause.eu/), in which causative mechanisms and molecular pathways linking early life stress to depression and two of its main physical comorbidities, coronary heart disease and diabetes, will be identified. This work will see the construction of a specific data portal and support data workflows as part of ENA’s data coordination workflow and bring new cell line and microbiome data into the ENA. In the marine scope, BlueCloud (https://www.blue-cloud.org/), an EU project to build a thematic European Open Science Cloud for the aquatic domain, has been initiated, in which we provide ENA services as data sources/destinations into the project's Virtual Research Environments and will develop a plankton genomics demonstrator platform. This work is in close synergy with the newly awarded EU AtlantECO that began in the latter half of 2020. In the biodiversity genomics area we are developing a data tracking system and public data portal for the Darwin Tree of Life project, in which all ∼66 000 eukaryotic species from the UK and Ireland will be sequenced and analysed at the genomic level. This work will share technical elements with the GBMF-funded Aquatic Symbiosis Genomics project, newly started in 2020. Sample checklists have been implemented for both these projects to capture high quality metadata enabling findability and reuse of data. The EU ELIXIR-CONVERGE project (https://elixir-europe.org/about-us/how-funded/eu-projects/converge), launched in January 2020, brings the opportunity to explore different data submissions brokering models; a recent call for participation resulted in significant interest. Along with the ongoing EASI-Genomics project (https://www.easi-genomics.eu/home) in which we will set up submissions brokering with European sequencing facilities, this work furthers our goals towards a broader data submissions brokering network for ENA.

## DATA AVAILABILITY

ENA services are freely available at (http://www.ebi.ac.uk/ena). Content is distributed under the EMBL-EBI Terms of Use available at (https://www.ebi.ac.uk/about/terms-of-use).
